# The physiological response of the Arctic key species Polar cod, *Boreogadus saida*, to hypoxia in a warming ocean: critical oxygen levels and swimming performance

**DOI:** 10.1186/s40850-025-00241-3

**Published:** 2025-10-11

**Authors:** Sarah Kempf, Carolin Julie Neven, Alexander Timpe, Félix P. Leiva, Brad Seibel, Felix Christopher Mark

**Affiliations:** 1 Integrative Ecophysiology, Alfred Wegener Institute Helmholtz Centre for Polar and Marine Research, Am Handelshafen 12, 27570 Bremerhaven, Germany; 2 University of Bremen, Bibliothekstraße 1, 28359 Bremen, Germany; 3 Ifremer, HMMN, Laboratoire Ressources Halieutiques, 150 Quai Gambetta, Boulogne-sur-Mer, 62200 France; 4 College of Marine Science, University of South Florida, St Petersburg, FL 33701 USA; 5 Department of Environmental Science, Radboud University Nijmegen, Nijmegen, GL 6500 The Netherlands

**Keywords:** Hypoxia, Ocean warming, Metabolic scope, *P*_crit_, Swimming performance, Polar cod

## Abstract

Global warming has already caused a loss of nearly 50% Arctic sea-ice coverage since the 1980s. This sea-ice loss strengthens summer stratification of the ocean’s water column and, consequently, hypoxic zones may form in the deep-water layers. Our study investigated the response of an Arctic keystone species, the Polar cod, *Boreogadus saida*, to hypoxia and warming. We measured the metabolic capacity (standard, routine and maximum metabolic rates, aerobic scope, and critical oxygen saturations (*P*_crit_ and *P*_cmax_)) and swimming performance of Polar cod under progressive hypoxia at 2 °C and after warm acclimation at 10 °C using intermittent-flow and swim tunnel respirometry. We observed stable patterns that were similar in both temperatures: Polar cod displayed oxygen-regulating behaviour under progressive hypoxia, with standard metabolic rates never below aerobic baseline metabolism at 2 °C and a very stable aerobic scope for both temperatures. Our study revealed that Polar cod can handle oxygen saturations down to a *P*_crit_ of 12.56 ± 1.59% air saturation at typical habitat temperatures. At 10 °C, *P*_crit_ rose to 24.37 ± 1.65% air saturation. We suggest that low-oxygen areas could potentially serve as protective zones for Polar cod for the next decades as long as bottom water temperatures still remain cold in summer. However, since hypoxia tolerance will decrease with warmer temperatures, this physiological advantage over invading species and predators will be progressively lost.

**Supplementary Information:**

The online version contains supplementary material available at 10.1186/s40850-025-00241-3.

## Introduction

Arctic ecosystems are characterized by strong seasonal fluctuations in sea ice coverage and formation. The ongoing climate change is leading to a rapid warming of the Arctic, and due to Arctic amplification, the Arctic ocean is expected to warm twice as fast as the global average [1, 2]. Due to increased sea surface temperatures, the Arctic has already lost 49% of its sea ice, compared to the 1979–2000 baseline of 7.0 × 10^6^ km^2^ [3, 4] and, according to a business-as-usual greenhouse gas emission scenario (RCP8.5) [5–7], it is projected that the Arctic will be nearly ice-free in September before the year 2050 [8]. 

This dramatic sea-ice loss has also been observed in the fjord systems of the Svalbard archipelago in the Arctic ocean (74° to 81° N, 10° to 35° E) [9–11]. While some of the Svalbard fjords are widely open to the neighbouring sea (e.g. Kongsfjorden, Isfjorden), others are not and can be regarded as closed, or semi-closed, due to their relatively shallow sills (e.g.: van Mijenfjorden, Billefjorden, Brepollen/Hornsund) [11–13]. In these systems, the loss of sea ice and declining sea surface salinities, due to freshwater inflow, can lead to increased upper ocean stratification. In stagnant deep-water bodies, this will result in oxygen depletion by biological processes, which can pose a challenge for marine organisms. In semi-closed fjord systems, the thermohaline circulation caused by sea ice formation is the main driver of the abiotic conditions in the deep-water layers [14]. The seasonal downward transport of cold, dense and highly saline water from the surface to the bottom breaks up summer stratification and thereby replenishes oxygen and nutrient levels in the bottom waters with locally formed winter cooled water (WCW) [13, 15]. The reduced ice formation or even a complete lack of ice cover is having serious ecological consequences as the deep cold-water layers are not receiving sufficient amounts of oxygen-rich water and oxygen depletion may extend over more than one season [16, 17]. The replacement of WCW by local- and intermediate water in warmer years, as demonstrated for Hornsund in Svalbard [13], can potentially lead to local bottom hypoxia.

**Fig. 1:**
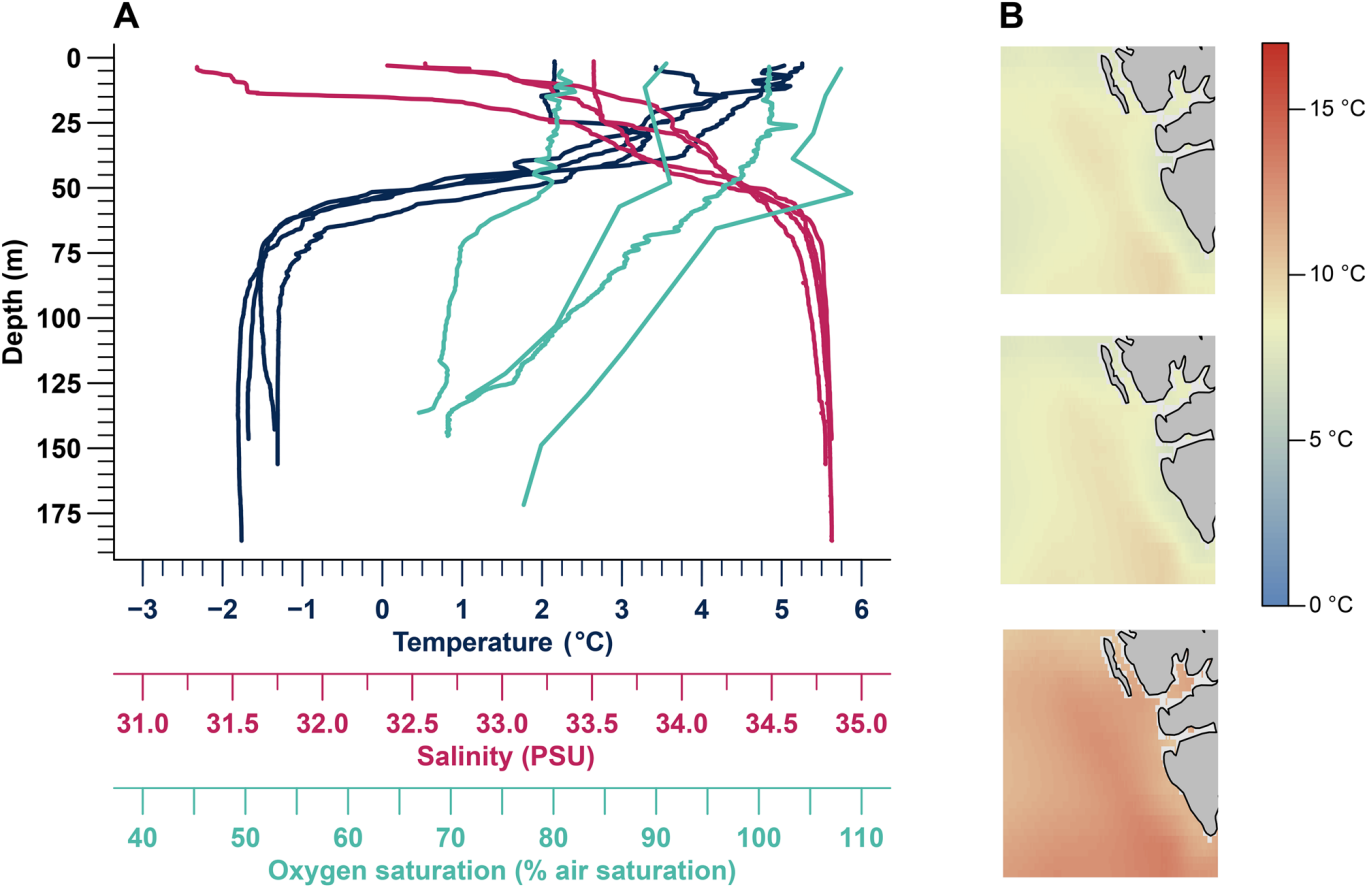
CTD profiles (2013–2020) and sea surface temperature models for 2100 of the study area. **A**: vertical profiles of temperature (dark blue, in °C), salinity (pink, in PSU), and oxygen (turquoise, in % air saturation) in Billefjorden. CTD profiles casts were conducted in the years 2013, 2015, 2018 and 2020 during RV Heincke cruises HE408, HE451, HE519 and HE560 [data from 18–21]. Due to technical issues with the oxygen sensor, only a limited number of oxygen saturation readings were recorded during the 2015 CTD cast. In 2020, the oxygen saturation was measured manually after the CTD cast using an optode. **B**: projected sea surface temperature for the year 2100 along the west coast of Svalbard under three scenarios: RCP 4.5, RCP 6.0 and RCP 8.5. These projections were modelled in R using BioOracle data, based on the IPCC (2014) scenarios [5]

In Billefjorden, oxygen levels regularly decrease from 100% air saturation (366.67 µmol O_2_/L) in top water layers down to 75% air saturation (275.01 µmol O_2_/L) at the bottom, at the end of summer (Fig. 1A). If WCW formation does not occur in an ice-free winter, oxygen levels may fall far below these values in the following summer.

One of the most abundant fish species inhabiting these deep fjord basins is Polar cod (*Boreogadus saida*). This species plays a key role in the Arctic ecosystem, serving as a key link between lower trophic levels and top predators [22]. Polar cod feed on small invertebrates such as copepods, amphipods, gastropods and krill, and are themselves a main food source for large fish, marine birds, and mammals [reviewed by 23]. The habitat of *B. saida* varies depending on its life stage. Young-of-the-year individuals are typically found in relatively warm surface and midwater layers (up to 6 °C, pers. obs. FC Mark) during their first summer, while juveniles are often associated with sea ice. Adult Polar cod spend the winter seasons in both pelagic and bottom waters, and retreat to cold WCW layers at the fjord bottom during summer [cf. 23]. These isolated fjord basins, with temperatures as low as −1.9 °C (Fig. 1A) provide protection for Polar cod from warming and predation by invading species such as Atlantic cod, *Gadus morhua* [24–27]. Given their key role in the Arctic food chain, recent studies have focused on the ecology, physiology, and resilience of *B. saida* to climate change [e.g. 22, 28–34]. In the light of the ongoing transformation and Atlantification of the Arctic ecosystem around Svalbard [35], and potential future deoxygenation of its deep fjords, this study assessed the hypoxia tolerance and metabolic performance under progressive hypoxia at 2 °C and after long-term (4 months) acclimation to 10 °C. The latter temperature is close to Polar cod’s critical temperature (T_crit_) of 12 °C, above which long-term mortality increases sharply (pers. obs. FC Mark). We focused on the standard (SMR), active (AMR), maximum metabolic rate (MMR) and aerobic scope (AS) to evaluate how progressive hypoxia influences *B. saida*’s metabolic performance traits, and to identify its critical oxygen concentration (*P*_crit_), which is the oxygen partial pressure below which the metabolic rate of an organism begins to be dependent on oxygen concentration, coupled with an increasing contribution of anaerobic metabolism [cf. 36–39]. We further analysed their critical oxygen concentration for maximum metabolism (*P*_cmax_), swimming performance to determine the *P*O_2_ dependence of U_gait_, the speed at which the fish transitioned from a steady to a “kick-and-glide” swimming gait (which is considered to be anaerobically supplemented), and U_crit_, the maximum swimming velocity achieved in the swim trials [cf. 29].

Hypoxia tolerance has been scarcely investigated in Polar cod, partly owing to the assumption that polar ectotherms have not evolved this trait in typically oxygen-rich cold polar waters [40–43]. To address this knowledge gap, and predict the consequences of future climate change scenarios in these already strongly affected ecosystems, we need to understand the physiological response of this Arctic key species to local hypoxia and rising temperatures [44–46].

## Materials and methods

### Fish collection and husbandry

Polar cod (*Boreogadus saida*) were caught in October 2018 during RV Heincke expedition HE519 in Billefjorden (78° 34’ 59.99” N; 16° 27’ 59.99” E), the innermost part of Isfjorden, located on the west coast of the Svalbard archipelago. The fish were caught using a fish-lift connected to a pelagic trawl at 150 m depth [47]. In situ temperature was −1.5 °C, and the oxygen level was at 75% air saturation (275.01 µmol O_2_/L).

The fish were transported to the Alfred Wegener Institute (AWI) in Bremerhaven, Germany, where they were kept in two recirculating rearing tanks at an ambient temperature of 0.0 ± 0.5 °C and above 95% air saturation (20 kPa). For 12 months fish were fed to satiation twice a week with commercial pellet food (Skretting, Norway) and frozen squid.

Following this period, 46 adult fish were anaesthetized with tricaine methanesulfonate (MS-222; 0.125 g L^−1^) and tagged with passive glass transponders (PIT tag, FDX-B, 7 × 1.35 mm, Loligo Systems Denmark) near the dorsal fin. During this procedure, fish were also measured (total and standard length, width and depth) and weighed. No subsequent mortality due to tagging and handling stress occurred.

### Thermal acclimation

The 46 fish were divided into two groups. The first group (*n* = 30; cold acclimated, C) had an average total length (± SD) of 19.7 ± 1.3 cm and total weight (± SD) of 39.6 ± 9.5 g. These fish were acclimated in the previously described aquarium tanks to 2.0 ± 0.3 °C over four months. The second group (*n* = 16; warm acclimated, WA) had a similar total length of 19.8 ± 1.9 cm and a total weight of 41.3 ± 10.1 g. This group was progressively acclimated with a warming rate of 0.75 °C every second week. Reaching 12 °C, increased mortality occurred and the acclimation temperature was decreased to 10 °C at which they were maintained for four months prior to the experiments. The temperature of 10 °C is thus close to the critical temperature of Polar cod, yet within the thermal range of the projected future surface water temperatures (see Fig. 1B) for the western fjord systems of Svalbard according to RCP 8.5 [5].

Experimental use of the 2 °C group individuals was randomised, resulting in up to four different *P*O_2_ levels per individual (for further information see Table S4 in the supplements). Following the experimental trials, the fish were maintained at normoxia to allow for recovery for one week. Fish that were used more frequently in the 2 °C experiments did not show significantly higher hypoxia tolerance than others (*p* = 0.2, tested by a Wilcoxon rank-sum test to compare the distributions of hypoxia tolerance between the usage frequencies). For the WA group, individuals were used repeatedly for every *P*O_2_ level unless mortality occurred (see Table S5 in the supplements). For both temperatures, the replacement of some individuals within the trial or the exclusion of data from specific individuals from later analysis was necessary due to mortality.

### Metabolic rates under progressive hypoxia

For the first experimental group (C), an experimental temperature of 2 °C was chosen for respiration chambers and swim tunnel, according to temperatures previously used for Polar cod to determine metabolic rate, mitochondrial performance and growth [28, 31]. The second group (WA) was measured at their acclimation temperature of 10 °C to gauge the remaining aerobic scope close to T_crit_.

Routine metabolic rates (RMR) were measured using seven fully automated respiration chambers (Loligo Systems ApS, Denmark), submerged in two connected thermoregulated tanks (170 L), and used to calculate the standard metabolic rates (SMR). The water from the outer reservoirs was re-circulated to each respirometer using computer-controlled flush pumps (Compact 600, EHEIM, Germany), relays and a software (AutoResp 2.3.0, Loligo Systems ApS, Denmark). Each respirometry chamber also had its own circulation loop including an oxygen sensor (Witrox 4 oxygen meter, Loligo systems ApS, Denmark) that continuously monitored water oxygen level inside the chamber. Oxygen probes were calibrated to 0% air sat. (nitrogen saturated water) and 100% air sat. (fully aerated water) at the beginning of each respirometry trial.

Oxygen consumption ($\dot M$O_2_) measurement cycles consisted of an open period, during which respirometers were replenished with water from the surrounding reservoir, and a sealed period, when the flushing pumps were turned off and the decline in water oxygen over time was used to calculate the corresponding fish $\dot M$O_2_. Note that the first 200 s of each measurement cycle was not used for the calculation of $\dot M$O_2_ (stabilization period). The respirometers’ measurement cycles were 5 min flush, followed by 30.5 min measurement in the cold group (C) and 2.5 min flush, followed by 17.5 min measurement in the WA group. The respirometry set-up was located in a space with restricted access and visual contact between the animals was prohibited by opaque plastic walls between the chambers. Note that fish were fasted 7 days (group C) or 3 days (group WA) prior to being transferred to the respirometers. This starvation period was chosen according to Kunz et al. [48], who found specific dynamic action (postprandially elevated $\dot M$O_2_, SDA) to last 167 h at 0 °C and 135 h at 6 °C, and calculations of gastric evacuation halftime (146 h at −0.49 °C) by Hop and Tonn [49].

**Fig. 2:**
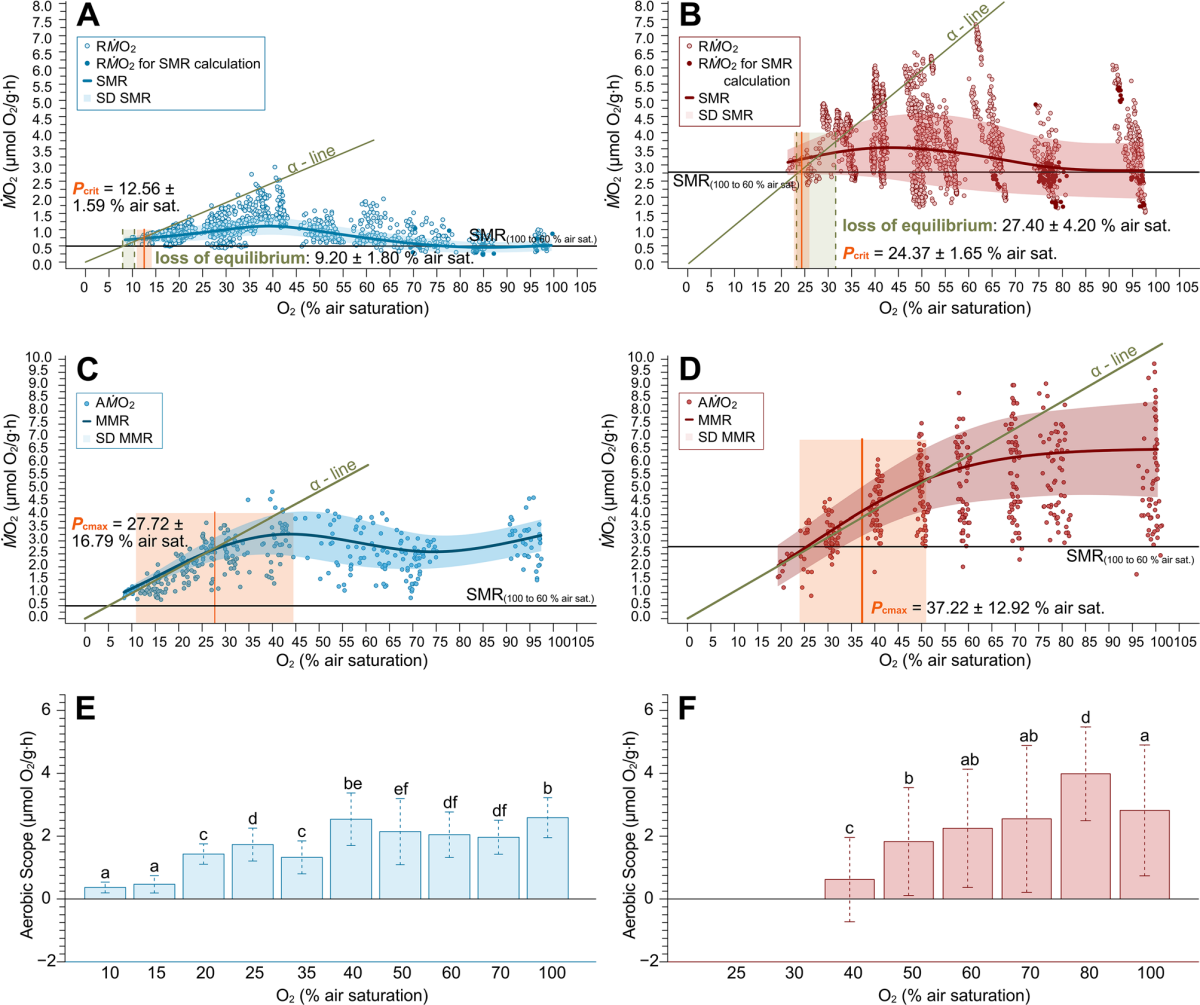
Routine, and active metabolism; standard, and maximum metabolic rate and corresponding aerobic scope under progressive hypoxia at 2 and 10 °C. The oxygen consumption ($\dot M$O_2_ in µmol O_2_/g∙h) and aerobic scope (AS in µmol O_2_/g∙h) over oxygen saturation (in % air saturation) are displayed for both experimental temperatures. **A** & **B**: routine metabolic rate (circles, R$\dot M$O_2_) and standard metabolic rate (line, SMR as polynomial regression) with standard deviation (shaded area, SD SMR) and temperature specific SMR (black line; A: 0.49 ± 0.20 µmol O_2_/g∙h, B: 2.78 ± 0.87 µmol O_2_/g∙h), complemented by the indication of the critical oxygen saturations (orange line, mean *P*_crit_ ± SD) and α-line for 2 °C (A) and 10 °C (B). At the lower end of the oxygen range, fish lost equilibrium (green shaded area), which terminated the experiment (A:1887 single $\dot M$O_2_ data points representing 86 experimental runs of 30 individuals (*n* = 7 per *P*O_2_ level), B: 2473 single $\dot M$O_2_ data points representing 53 experimental runs of 23 individuals (*n* = 7 per *P*O_2_ level). **C** & **D**: active oxygen consumption (circles, A$\dot M$O_2_) and maximum metabolic rate (line, MMR as polynomial regression) with standard deviation (shaded area, SD MMR) complemented by the indication of the critical oxygen saturations (orange line, mean *P*_cmax_ ± SD) and α-line for 2 °C (A) and 10 °C (B). As in A and B, the temperature specific SMR is included as black line (C: 361 single $\dot M$O_2_ data points representing 60 experimental runs of 30 individuals (*n* = 6 per *P*O_2_ level), D: 431 single $\dot M$O_2_ data points representing 58 experimental runs of 23 individuals (*n* = 7 per *P*O_2_ level)). **E** & **F**: AS (in µmol O_2_/g∙h) at 2 °C (E) and 10 °C (F) across *P*O_2_ levels (in % air saturation) are shown as bar plots with standard deviation. Identical letters indicate no significant difference (*p* > 0.05) between *P*O_2_ levels, based on results of pairwise t-tests

Each oxygen saturation was maintained for two days and two nights, containing approximately 80–100 measurement phases during which fish were left undisturbed to ensure proper determination of the standard metabolic rate (SMR) as they habituated to the experimental conditions, yet only the last night (dark phase) of measurements was used for subsequent calculation of $\dot M$O_2_ [50]. Oxygen consumption was measured at 12 different *P*O_2_ levels at 2 °C, 100, 85, 75, 65, 55, 40, 35, 30, 25, 20, 15, and 10% air saturation, and eight different *P*O_2_ levels at 10 °C, 100, 75, 65, 55, 50, 40, 30, 25% air saturation (*n* = 7 per *P*O_2_ level for both temperatures). The desired *P*O_2_ was achieved by changes in the amount of nitrogen gas (N_2_) bubbled into the water by a feedback-controlled solenoid valve (AutoResp software version 2.3.0, Loligo Systems ApS, Denmark), while the aeration with compressed air was kept constant throughout the experiments. Moreover, the measurement was terminated prematurely upon the observation of the individual’s inability to right themselves [according to 51]. In this case, the corresponding *P*O_2_ was documented as “loss of equilibrium” (see Fig. 2).

#### Oxygen consumption ($\dot M$O_2_) calculation

The rates of oxygen consumption were calculated after Boutilier et al. [52] and normalized according to Steffensen et al. [53] using the statistical language “R” with the packages “FishResp” [54] and “Mclust” [55]. At both temperatures, oxygen consumption was elevated upon the introduction of fish in the respirometry chambers, but progressively declined to a steady level after about 36 h. Therefore, we only used $\dot M$O_2_ data of the second night to calculate routine and standard metabolic rates.

$\dot M$O_2_ and the corresponding mass-specific metabolic rates for both experiments were calculated from optode oxygen recordings (1 Hz) measured in the respiration chambers and swim tunnel, and normalized in R [56] using the package “FishResp” [57]. Afterwards, $\dot M$O_2_ was normalized to a common bodyweight (BW) of 100 g according to Steffensen et al. [53] using the following equation

$$\dot M{\rm{O}_{2(100)}} = \dot M{{\rm{O}}_{\rm{2}}}\, \cdot \,{\left( {{\rm{BW/100}}} \right)^{{\rm{(1 - 0}}{\rm{.8)}}}}^{}$$

with a scaling exponent of 0.8 according to Killen et al. [b = 0.83 for *Boreogadus saida* 58]

After normalization, we calculated a polynomial regression of $\dot M$O_2_ over *P*O_2_ using the “smooth.spline” function from the R package “stats” [version 4.4.0 59], the number of terms was determined by penalized log likelihood.

For both temperatures, bacterial respiration was measured after the respirometry trials and accounted for less than 2% of the fish’s respiration. The data were corrected for this background respiration using the “empty.chamber” method for 2 °C, and the “post.test” method for the 10 °C data of the “FishResp” package in R. All chambers were cleaned weekly.

### Swimming performance under progressive hypoxia

The metabolic rate and swimming performance of *B. saida* under hypoxia were recorded following a critical swimming speed (U_crit_) protocol (Kunz 29, modified after Brett, 61), applying 10 different *P*O_2_ steps at 2 °C (100, 75, 65, 55, 40, 30, 25, 20, 15 and 10% air saturation; *n* = 6 per *P*O_2_ level) and nine different *P*O_2_ levels at 10 °C (100, 75, 65, 55, 50, 40, 30, 25 and 20% air saturation; *n* = 7 per *P*O_2_ level).

A Brett-type swim tunnel respirometer of 5 L (test section 28 × 7.5 × 7.5 cm, Loligo Systems ApS, Denmark) was used to measure the swimming performance of *B. saida* (*n* = 6–7 per *P*O_2_ level). Oxygen content and flush phases were controlled by a DAQ-M instrument (Loligo Systems ApS, Denmark). The swim tunnel was submerged in a reservoir tank to maintain stable abiotic conditions within the chamber. The water velocity was adjusted by a control unit regulating the revolutions/min of a motor and connected propeller (Loligo Systems ApS, Denmark). To calibrate the water velocity to voltage output from the control system, a vane wheel flow probe (Loligo Systems ApS, Denmark) was used. The *P*O_2_ was determined using fiber optic mini sensors (optodes) connected to a four-channel oxygen meter (Witrox 4 oxygen meter for mini sensors, Loligo Systems ApS, Denmark).

As for SMR measurements, the fish were transferred to the swim tunnel after seven days (C) or three (WA) days of fasting, in water-filled bags to minimize handling stress, and to keep the air exposure to a minimum. After an acclimatisation period of 1.5 h in stagnant water, water velocity was increased to 1.2 BL (body length)/sec for 25 min. Afterwards, the velocity was increased to the first measurement velocity of 1.4 BL/sec before starting the U_crit_ protocol. Each velocity step contained an $\dot M$O_2_ measurement cycle, comprising a 60 s flush phase followed by 120 s of stabilisation and an 8 min measuring period, after which water velocity was increased by 0.15 BL/sec. Each measurement period thus returned 480 oxygen measurements from which oxygen consumption rate ($\dot M$O_2_) was calculated. The swim chamber was covered to minimise disturbance.

This stepwise increase was performed until exhaustion, when the fish completely refused to swim and remained inactive for more than 90 seconds. The maximum metabolic rate was determined for each *P*O_2_ level inside the swim tunnel.

To determine the gait-transition speed U_gait_ (the switch from strictly aerobic to anaerobically supplemented swimming) [54], kick-and-glide swimming (so called “bursts”) [60] was documented. In kick-and-glide swimming, thrust generation is supplemented by anaerobic muscle contractions of mainly the white muscles. All bursts were counted and the corresponding time of kick-and-glide swimming during the swim trial was documented. The critical swimming velocities (U_crit_) of the fish were calculated according to Brett [61]. After U_crit_ was reached, the velocity was immediately decreased to the basic settling velocity of 1.4 BL/sec and the fish stayed in the swim tunnel for another 10 min before being transferred back into their tanks.

Blank respiration in the swim tunnel accounted for less than 2% $\dot M$O_2_. The swim tunnel was cleaned daily.

#### Critical swimming speed (U_crit_) after Brett (1964)

The critical swimming speed was calculated as:

$${U_{crit}} = {U_{max}} + {{vT} \over t}$$

With U_max_ as the highest water speed (v) that the fish were able to perpetuate for a complete time interval (t) and T as the time spent at the given velocity (U_crit_) until exhaustion of the fish.

#### Gait transition speed (U_gait_)

The critical swimming speed was calculated as:

$${U_{gait}} = {U_{steady}} + {{vT} \over t}$$

with U_steady_ as the highest water speed (v) perpetuated for a complete time interval (t) without burst swimming and T as the time spent at the given velocity leading to burst/gait swimming.

### Physiological indices and their calculation

#### SMR and MMR

The standard metabolic rate (SMR) represents the minimum rate of oxygen consumption that is observed when an organism is at rest and in a post-absorptive state, whereas the maximum metabolic rate (MMR) represents the highest rate of oxygen consumption that an organism is capable of achieving [62, 63]. This concept is crucial for understanding an organism’s capacity for aerobic activity, growth, and reproduction under varying environmental conditions. MMR is generally determined by measuring oxygen consumption under exhausting exercise. For fish, this can be achieved by applying a critical swimming speed (U_crit_) protocol (see above 2.4). U_crit_ can be experimentally detected under changing environmental conditions to examine their influence on activity and MMR [61, 64–66].

The SMR was calculated as the 20% quantile of the entire dataset, after Chabot et al. [50], using the function “quantile” of the R package “rstatix” (version 0.7.2.) [55]. Briefly, the lowest 5% of the data obtained from the respiration chambers were removed as outliers, afterwards the lowest 15% quantile of the remaining values was calculated [50]. The lowest 15% quantile of the $\dot M$O_2_ of each fish of the oxygen range between 100% and 60% air saturation were averaged and defined as the temperature specific SMR for each treatment. We also calculated *P*O_2_ specific SMRs for both temperature treatments. For each oxygen saturation, the lowest 15% quantile of each individual’s $\dot M$O_2_ was determined and the mean defined as the SMR for the respective *P*O_2_ levels.

For MMR calculations, we used the reverse approach of Chabot et al. [50] and discarded the highest 5% of the data from the swim trials (≙ AMR) as outliers and then, different from the SMR calculation and due to the lower number of total MR per individual, calculated the mean of the remaining highest 30% quantile. Following the approach of the above-mentioned oxygen-specific SMRs per temperature treatment, we calculated the MMR for each oxygen saturation.

#### Aerobic scope

The capacity for aerobic activity is determined by the aerobic scope (AS) [62], the difference between maximum- and standard oxygen consumption rate [63].

Based on the individual SMR and MMR data, the AS and factorial AS (FAS) per *P*O_2_ group was calculated in R using the package “tidyr” [67] as follows:

$$\text{AS}=\text{MMR}-\text{SMR};\quad\text{FAS}=\text{MMR/SMR}$$

#### ${P_{crit}},{P_{cmax}}$ and the maximum oxygen supply capacity

The minimum oxygen level required to maintain standard metabolism has various names, e.g. the “level of no excess activity” [68], “*P*_crit_ (P_c_)” [69], or “O_2crit_” [70]. It is often referred to as the oxygen threshold below which fishes switch to anaerobic metabolism, with survival dependent on the specific oxygen debt defined by how far below SMR metabolism is suppressed and how long the situation persists [37, 70]. Below *P*_crit_, oxygen shifts from a limiting factor to a lethal factor. It is traditionally calculated from the point of intersection between MMR and the temperature specific SMR at declining oxygen, based on a regression analysis [cf. 71]. As we did not observe metabolic depression below SMR, we compared the traditional approach with the method recently published by Seibel et al. [72], which introduces the oxygen supply capacity alpha and quantifies the oxygen dependency of maximum metabolic rate by the so-called alpha line [72]. According to this method, the physiological capacity for oxygen supply (α_0_, µmol O_2_/g∙h∙ kPa) reaches its maximum (α) at *P*_crit_:

$${P_{crit}} = {{\dot M{O_2}} \over \alpha }$$

α can be calculated for any $\dot M$O_2_ between standard and maximum [according to 72] and corresponds to the lowest *P*O_2_ at which a given metabolic rate can be sustained. When derived from the SMR, this *P*O_2_ threshold is indicated as “*P*_crit_” or “*P*_crit-SMR_”. If determined from the MMR, it is indicated as “*P*_cmax_” or “*P*_crit-MMR_”, respectively. In this study, *P*_crit_ corresponds to *P*_crit-SMR_ and any other *P*_crit_ is further specified. According to this approach, α_0_ was first calculated as follows for each measured fish and measurement period:

$${\alpha _0} = {{\dot M{O_{2\left( {100} \right)}}} \over {P{O_2}}}$$

with the individual’s $\dot M$O_2_ in µmol O_2_/g∙h and *P*O_2_ during the measurement in kPa.

Afterwards, α was determined for each temperature treatment as the average of three highest α_0_ observations of each individual [according to 72]. Furthermore, the corresponding α-value served as the slope of a linear function, the so-called “α-line”. This line quantifies the oxygen dependency of MMR and the rate dependence of *P*_crit_.

*P*_cmax_, the *P*O_2_ at which oxygen supply fails to cover the maximum demand for oxygen resulting in a decrease of MMR [72–74], can be calculated as the intersection of a linear function [α-line 72] with a slope α = MR_*P*crit_/*P*_crit_ with MMR.

### Data correction and statistics and outlier analysis

#### Correction of calibration error

The oxygen supply (α_0_) for each measure period was calculated as the mass-standardized metabolic rate divided by the average *P*O_2_. The oxygen supply capacity (α) for each temperature treatment was determined as the average of three highest α_0_ observations. Some trials at 2 °C showed signs of oxygen probe calibration error and thus were corrected accordingly [75]. Calibration error can dramatically elevate calculations of α_0_ near anoxia and bias the α-line with a few outlier observations. Briefly, the correction method assumes that, if the effect of *P*O_2_ error were removed, a trial or dataset would contain multiple equivalent estimates of α. A small *P*O_2_ correction value (1.46 kPa ≙ 7% air sat.) was added to each datapoint in the 2 °C dataset to satisfy this condition and counteract the effect of calibration error.

#### Outlier analysis

We also performed an outlier analysis, using a boxplot method in R (“rstatix” version 0.7.2), to eliminate general artefacts, such as spontaneous activity, during SMR measurements. This method identifies two categories of outlier: (1) outliers and (2) extreme points. Values above the third quantile of the data (Q3) plus 1.5 times the interquantile range (IQR = Q3 - first quantile of the data (Q1)) or below Q1 – 1.5 × IQR are considered as outliers. Values above Q3 + 3 × IQR or below Q1 – 3 × IQR are considered as extremes, we removed both categories as outliers before further data analysis. The outlier analysis affected 23.73% of the data (401 single data points) during the SMR measurement at 2 °C, and 1.54% of the data (38 single data points) at 10 °C. Within the data obtained from the swim tunnel trials, 0.83% (3 single data points) of the data at 2 °C were identified as outliers and no data were identified as outliers at 10 °C by the outlier analysis.

#### Statistical analysis

For each experiment, data were tested for normal distribution using a Shapiro-Wilk test. Variance homogeneity was tested using Levene’s test. In case of normal distribution and homogeneity, the significant differences between *P*O_2_ levels were tested for by one-way ANOVA (respiration chambers, burst counts and active swimming duration) or two-way ANOVA (swim tunnel, after logarithmic transformation) followed by pairwise-t-tests with Benjamini-Hochberg adjustment of the p-values. Furthermore, the influence of body weight, body length, temperature (Kruskal-Wallis test) and water velocity (two-way ANOVA) were examined. The level of statistical significance was set to *p* < 0.05 for all statistical tests. All statistical tests were performed in R language (version: 4.2.2).

## Results

A summary table of the results and exact O_2_ saturations of the *P*O_2_ levels (Table S1), and a table with a conversion of *P*O_2_ saturation in different units (% air saturation, µmol/L, kPa) (Table S3) can be found in the appendix.

### Mortality

During the experiments at 2 °C, mortality (*n* = 2) occurred only during the settling phase at the lowest *P*O_2_ (10% air saturation). At 10 °C, two individuals died at 25% air saturation in the respiration chambers. Therefore, the measurements were prematurely terminated and SMR had to be calculated from the $\dot M$O_2_ data of the five remaining individuals. During the swim tunnel experiment, one individual died at the end of the settling period to 20% air saturation.

### Metabolic performance

$\dot M$O_2_ was significantly influenced by body weight (p_RMR_ < 0.0001, p_AMR_ < 0.01). Additionally, AMR (active $\dot M$O_2_) was also found to be significantly influenced (*p* < 0.01) by body length.

#### SMR

The temperature specific SMR of *B. saida* was calculated after Chabot et al. [50] based on the $\dot M$O_2_ data recorded between 60 and 100% air saturation. At 2 °C, it amounted to 0.49 ± 0.20 µmol O_2_/g∙h. After acclimation to 10 °C, SMR was significantly increased almost 6-fold, reaching 2.78 ± 0.87 µmol O_2_/g∙h (*p* < 0.001) (Fig. 2).

At both temperatures, $\dot M$O_2_ never decreased below its temperature specific SMR throughout the oxygen range (Fig. 2A& B).

At 2 °C, $\dot M$O_2_ was significantly dependent on *P*O_2_ below 35% air saturation (*p* < 0.001). SMR (Fig. 2A, Table S1) remained on a steady level between 100 and 55% air saturation (max: 0.74 ± 0.13, min: 0.40 ± 0.10 µmol O_2_/g∙h), below which oxygen consumption increased between up to 1.25 ± 0.19 µmol O_2_/g∙h (*p* < 0.001). After this significant increase at 35% air saturation, $\dot M$O_2_ decreased down to 0.88 ± 0.31 µmol O_2_/g∙h (*p* < 0.0001) and remained stable between 25 and 10% air saturation (0.88 ± 0.31 µmol O_2_/g∙h − 0.72 ± 0.01 µmol O_2_/g∙h).

SMR at 10 °C was significantly affected by progressive hypoxia below 50% air saturation (*p* < 0.001), and also by the elevated temperature when compared to the SMR at 2 °C (*p* < 0.001) (Fig. 2B, Table S1). SMR significantly increased at 40% air saturation (3.87 ± 0.93 µmol O_2_/g∙h) and levelled off towards 25% air saturation (2.80 ± 0.32 µmol O_2_/g∙h).

#### MMR

The $\dot M$O_2_ during the swim tunnel experiment was significantly influenced by *P*O_2_ (below 25% air saturation at 2 °C, and below below 50% air saturation at 10 °C, *p* < 0.01 for both temperatures) and water velocity (*p* < 0.001) at both temperatures. The highest O_2_ at 2 °C was detected at 2.60 BL/sec with an average of 4.94 ± 0.80 µmol O_2_/g∙h (Figure S4, left). At 10 °C, metabolic rates increased up to a water velocity of 2.9 BL/sec with a mean O_2_ of 6.47 ± 1.49 µmol O_2_/g∙h (Figure S4, right). A growing number of fish refused to swim with increasing water velocities at both temperatures, resulting in only three individuals reaching the maximum swimming speed of 3.5 BL/sec.

At 2 °C, MMR (Fig. 2C) was maintained between 100 and 25% air saturation (max: 3.47 ± 0.83, min: 2.71 ± 0.43 µmol O_2_/g∙h), below which MMR significantly decreased in parallel with decreasing oxygen content until a $\dot M$O_2_ of 1.09 ± 0.08 µmol O_2_/g∙h was reached at 10% air saturation (*p* < 0.05). At 10 °C (Fig. 2D), MMR was significantly higher than at 2 °C (*p* < 0.0001). Between 100 and 50% air saturation, MMR was maintained (max: 6.48 ± 0.86, min: 5.53 ± 0.12 µmol O_2_/g∙h) and began to significantly decrease below 50% air saturation (*p* < 0.05), following an oxygen conforming pattern. MMR at 40, 30, 25 and 20% air saturation was significantly lower than between 100 to 50% air saturation. MMR matched SMR at 25.99% air saturation. During the settling period to 25–20% air saturation, four of the seven fish lost equilibrium and one fish died at the end of the settling period.

#### Aerobic scope

At 2 °C, AS decreased in a stepwise manner (100–40, 30–20, 15–10% air saturation, *p* < 0.0001). AS accounted for 2.28 ± 0.82 µmol O_2_/g∙h within an oxygen range of 100–40% air saturation. In comparison, AS between 30 and 20% air saturation decreased to 1.55 ± 0.46 µmol O_2_/g∙h. In the lowest *P*O_2_ groups 15 and 10% air saturation, also the lowest AS of 0.43 ± 0.24 µmol O_2_/g∙h was calculated. The highest value for aerobic scope, 2.59 ± 0.64 µmol O_2_/g∙h, was recorded at 100% air saturation.

At 10 °C, AS varied within the oxygen range of 100–50% air saturation (2.61 ± 2.04 µmol O_2_/g∙h). At 40% air saturation, AS significantly dropped to 0.62 ± 0.34 µmol O_2_/g∙h (*p* < 0.0001), reaching zero at 25.99% air saturation (Fig. 2F).

The warm AS was initially larger at 100% air saturation but started to decrease earlier (70% vs. 50% air saturation), meeting levels of cold AS around 50% air saturation and then running in parallel along an oxyconforming line. However, AS did not differ significantly between the two temperature treatments (*p* = 0.69).

#### Maximum oxygen supply capacity, ${P_{crit}}$ and ${P_{cmax}}$

**Fig. 3:**
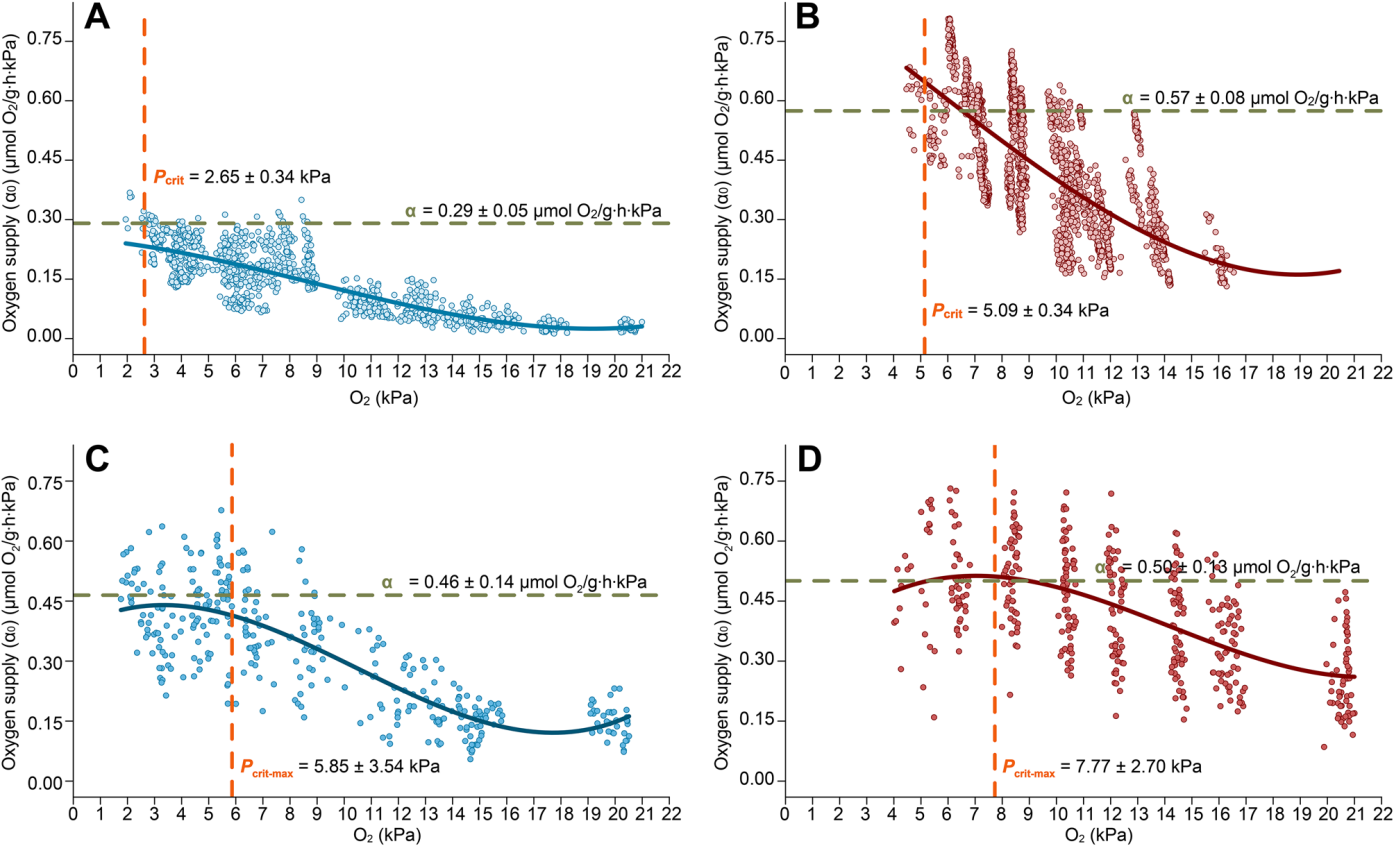
Oxygen supply (α_0_) under progressive hypoxia. The oxygen supply (α_0_, in µmol O_2_/g∙h∙kPa) over water *P*O_2_ (in kPa) is displayed for resting fish at 2 °C (**A**) and 10 °C (**B**), and active fish at 2 °C (**C**) and 10 °C (**D**). The oxygen supply capacity (α, in µmol O_2_/g∙h∙kPa) is indicated by a dashed green line and reaches its maximum at the critical *P*O_2_ (dashed orange line; *P*_crit_ for figures A and B, and *P*_cmax_ for figures C and D) [according to 72]. Values are given with standard deviation

At 2 °C, the maximum oxygen supply capacity, α, at rest accounted for 0.29 ± 0.05 µmol O_2_/g∙h∙kPa and resulted in a *P*_crit_ of 12.56 ± 1.59% air saturation (45.00 ± 3.53 μmol O_2_/L, 2.65 ± 0.34 kPa, Fig. 3A). Regression analysis resulted in a *P*_crit_ of 4% air saturation. After acclimation to 10 °C, α had significantly increased to 0.57 ± 0.001 µmol O_2_/g∙h∙kPa (*p* < 0.0001), which corresponds to a *P*_crit_ of 24.32 ± 1.65% air saturation (66.53 ± 2.48 μmol O_2_/L, 5.09 ± 0.34 kPa) (Fig. 3B), compared to 22% air saturation calculated using regression analysis.

At 2 °C, the maximum oxygen supply capacity during exercise accounted for 0.46 ± 0.14 µmol O_2_/g∙h∙kPa, indicating a *P*_cmax_ of 27.72 ± 16.79% air saturation (99.31 ± 16.91 µmol O_2_/L, 5.85 ± 3.54 kPa, Fig. 3C). After acclimation to 10 °C, α increased significantly to 0.50 ± 0.13 µmol O_2_/g∙h∙kPa (*p* < 0.05), which corresponds to a *P*_crit_ of 37.22 ± 12.92% air saturation (101.81 ± 12.69 µmol O_2_/L, 7.77 ± 2.70 kPa). In terms of oxygen content, α is thus identical under both temperature treatments (the higher % air saturation is due to the decreased oxygen solubility at increased temperature, Fig. 3D).

### Swimming performance

In general, we observed a decline of swimming activity with progressive hypoxia at both temperatures. At 2 °C, the total active swimming time significantly decreased with decreasing oxygen saturation (*p* < 0.05). Although we were not able to detect such a significant effect for the warm acclimated fish at 10 °C (*p* = 0.72), we found the lowest swimming activity at 25% air saturation. Furthermore, two fish lost equilibrium during the settling period to this *P*O_2_ level in the swim tunnel. At 20% air saturation, another two fish lost equilibrium during the settling period and one died.

#### Gait transition speed U_gait_

At 2 °C, *P*O_2_ had no significant influence on U_gait_ (*p* = 0.86).

Between 100 and 30% air saturation all, fish displayed burst swim behaviour and a rather constant U_gait_ of 2.25 BL/sec. Below 30% air saturation, the total occurrence of burst swim behaviour was reduced with progressive hypoxia (Table S1, mean burst counts per minute). At 20% air saturation, only three individuals displayed burst swim behaviour at all. The remaining three Polar cod stopped swimming without detectable burst actions. At 10% air saturation (U_gait_ = 1.87 BL/sec), only one Polar cod out of six displayed burst swim behaviour. Additionally, the burst swimming activity, determined as the total number of bursts (*p* < 0.05) and the number of bursts per minute (*p* = 0.05) decreased significantly with decreasing *P*O_2_ (Table S1). The maximum number of 2.9 ± 2.4 bursts per minute was achieved at 100% air saturation (Table S1).

Although statistical analysis revealed a global significant effect of *P*O_2_ on U_gait_ at 10 °C (*p* < 0.05), this was not confirmed by the post-hoc test (Wilcoxon-Test) between the individual *P*O_2_ levels. Generally, U_gait_ was relatively stable over a *P*O_2_ range of 100 to 65% air saturation (max: 2.1 BL/sec, min: 1.92 BL/sec) with a maximum of 2.05 ± 0.17 BL/sec at 65% air saturation (Table S1), yet significantly decreased compared to 2 °C (*p* < 0.001). The number of animals which showed burst swimming behaviour varied between the *P*O_2_ levels (100–30% air sat: *n* = 5–6) and decreased to an *n* = 2 at 25% air saturation. At 20% air sat, no more burst swimming behaviour occurred. The burst swimming activity (total number of bursts) and the number of bursts per minute did not reveal a significant *P*O_2_ effect (*p* = 0.23, and *p* = 0.42). Nonetheless, despite their variability, both parameters showed a decrease at 30% air saturation.

#### Critical swimming speed (U_crit_)

**Fig. 4:**
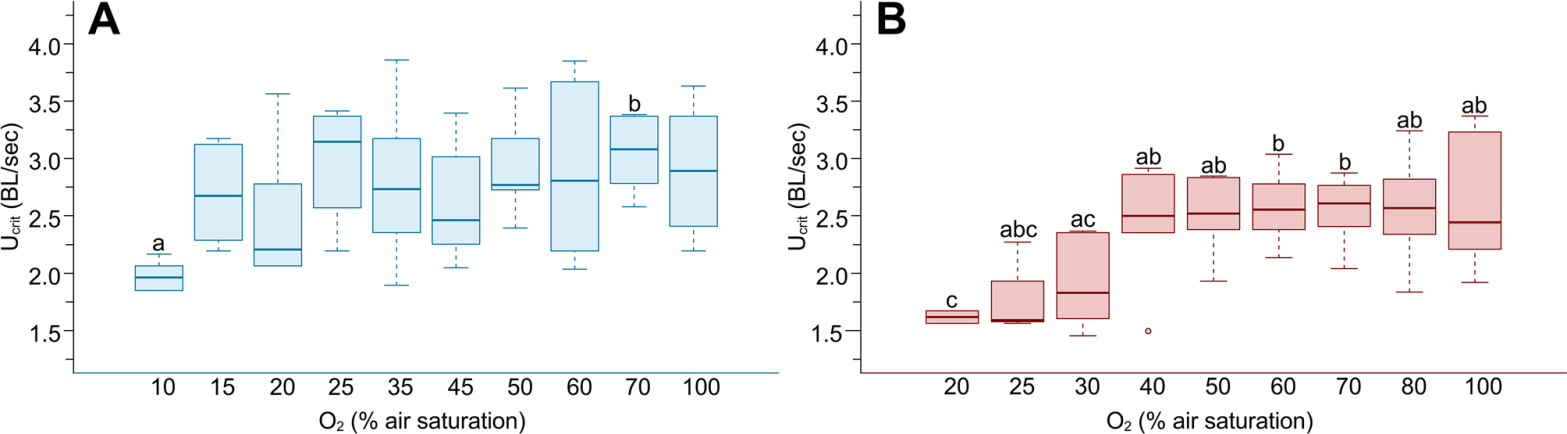
Critical swimming speed (U_crit_) under progressive hypoxia. Boxplots for comparing U_crit_ (in BL/sec) at different oxygen levels (in % air saturation) and two temperatures. (A) 2 °C, n = 6 per *P*O_2_ level; (B) 10 °C, n = 7 per *P*O_2_ level. Identical letters indicate no significant difference (*p* >0.05) among *P*O_2_ levels based on results of pairwise t-test

At 2 °C, U_crit_ (Fig. 4A) was maintained throughout all *P*O_2_ levels, only the U_crit_ values at 10% air saturation were significantly lower than at 75% air saturation (*p* = 0.008).

At 10 °C, U_crit_ (Fig. 4B) remained relatively stable over an O_2_ range from 100 to 40% air saturation, with significant differences between the lowest oxygen saturation (20% air saturation) and between 100 and 40% air saturation (*p* < 0.05). Generally, U_crit_ in the warmth was significantly lower than at 2 °C (*p* < 0.001).

Interestingly, U_crit_ (Fig. 4) remained constant over a far wider *P*O_2_ range than AS (Fig. 2E&F), for both temperatures. It significantly decreased at 10% air saturation in the cold treatment, and below 30–20% air saturation in the warm treatment, respectively.

## Discussion

### P_crit_ & P_cmax_

There are very few studies that have determined the critical oxygen saturatios and examined the influence of ambient oxygen levels on the aerobic scope of polar fish [e.g. 53, 76]. Over the whole range of their SMR and in part also for MMR (above 25% and 40% air saturation, respectively), Polar cod displayed oxygen regulating behaviour under progressive hypoxia, so that $\dot M$O_2_ never fell below SMR. As a result, we found *P*_crit_ to be unexpectedly low at 12.56 ± 1.59% air saturation (45.00 ± 3.53 µmol O_2_/L, 2.65 ± 0.34 kPa) and AS to be maintained in hypoxia until about 40% air saturation at typical habitat temperatures. This tolerance of Polar cod to lower oxygen saturations is certainly supported by their naturally very low SMR at 2 °C (0.49 ± 0.20 µmol O_2_/g∙h), indicating that these fish do not need much oxygen to sustain basic metabolic functions in their cold and so far thermally relatively stable habitat. This may have set the evolutionary basis for their apparent “hypoxia tolerance”, as similar energy conservation mechanisms can be used under both hypoxia and hypothermia [77–79]. Due to higher metabolic rates and lower O_2_ solubility closer to critical temperatures (10 °C) after warm acclimation, aerobic scope could only be maintained down to about 65% air saturation and *P*_crit_ rose to 24.37 ± 1.65% air saturation (66.53 ± 2.48 µmol O_2_/L, 5.09 ± 0.34 kPa, calculated with the α-method). Literature values for Greenland cod (*Gadus ogac*) and Atlantic cod (*Gadus morhua*) report temperature and life stage dependent *P*_crit_ ranging from 32% to 42% air saturation at 1 °C for adult Greenland cod, and 16.5–28.5% (at 5°C) to 15.5% (at 10°C) for juvenile Atlantic cod [22, 53, 74, 80, 81]. In Polar cod, a *P*_crit_ of 22–29% air saturation (59–79 µmol O_2_/L) at 10 °C is surprisingly similar to that of its larger relative, the Arctic-invading Atlantic cod [22, 83, 84], at the same temperature: 23% air saturation (63.29 µmol O_2_/L) [22, 80, 82–84]. Thus, in a warmer future, Polar cod would lose their advantage of a substantially lower *P*_crit_ over invading species and predators.

The methodological comparison between the intercept and α-methods resulted in lower critical oxygen thresholds: 4% and 22% air saturation with the intercept method at 2 and 10 °C respectively, indicating the general reliability of the α-method, which provides somewhat higher and potentially more realistic values for *P*_crit_. We also used the α-method to calculate *P*_cmax_, which was identical for both long-term acclimated groups (99–101 µmol O_2_/L, corresponding to 27.72 ± 16.79 and 37.22 ± 12.92% air saturation at 2 and 10 °C, respectively). This corroborates the notion that *P*_cmax_ is only dependent on oxygen concentration but independent of temperature [85]. Whether a constant *P*_cmax_ and increased MMR at 10 °C can be attributed to warm acclimation cannot be answered here (lack of acute 10 °C treatment), yet it is not uncommon to find acclimation effects at least in *P*_crit_ [e.g. 86]. Furthermore, although *P*_cmax_ was found to be relatively stable, it can be predicted that this threshold will increase in line with rising temperatures. This will result in a reduction of the physiological advantage of Polar cod over invasive species and predators. As temperatures rise further, Polar cod may struggle to maintain its metabolic efficiency, which is critical for survival, growth and reproduction [70, 87, 88]. However, it has been demonstrated that this critical temperature [8–10 °C, see also 89] is considerably higher than the projected water temperatures for the Arctic region for the next 80 years [cf. 90]. It can therefore be assumed that Polar cod are likely to encounter these conditions individually, but probably never in combination. This offers a hopeful perspective for this keystone species, indicating that they may still find refuge in the colder deep-water layers of the increasingly stratified fjords, even in the face of future warming. However, it is important to note here that we used adult fish in our experiments and that the conclusions drawn are based on the responses observed in this specific life stage. It is acknowledged that different life stages of Polar cod may exhibit varying degrees of sensitivity to oxygen limitation, with early developmental stages and spawners potentially showing a greater vulnerability to hypoxic conditions [cf 91, 92], so that successful reproduction appears unlikely under these circumstances. A further technical limitation of the present study may be caused by the lack of corresponding standard and maximum metabolic rates for each individual within each *P*O_2_ treatment, resulting in more variable but still significant effect sizes.

### Metabolic regulation

At 10 °C the SMR increased by a factor of 5 to 2.78 ± 0.87 µmol O_2_/g∙h (0.046 ± 0.001 µmol O_2_/g∙min), indicating the increased oxygen demand with increasing temperature and no apparent metabolic compensation through acclimation. Their ability to increase metabolic rates at thermal maximum temperatures (10 °C) beyond an expected Q10 of 2–3, while maintaining aerobic metabolism down to very low oxygen levels, largely contributes to their resilience in hypoxic conditions. At both temperatures, the SMR values appeared to be lower than those suggested by literature [e.g. 29, 53, 93], possibly due to improved measurement techniques and methodological differences such as short acclimatization periods and small sample sizes. For example, the SMR at 2 °C presented in this study was 6 times lower compared to the values reported by Kunz et al. [29], 3.018 ± 0.252 µmol O_2_/g∙h, at 3 °C. This may have a methodological cause: prior to the main respirometry experiments, we conducted an acute pilot experiment to define *P*_crit_, in which fish were allowed to respire until the loss of equilibrium. This occurred after 5.5 ± 1.4 hours at 2 °C and 2.4 ± 0.5 hours at 10 °C. The measured routine metabolic rates (2.23 ± 0.27 µmol O_2_/g∙h at 2 °C and 5.016 ± 1.040 µmol O_2_/g∙h at 10 °C) were similar to the SMR reported by Kunz et al. [29], highlighting the need for longer acclimation periods to reach true SMR in an undisturbed environment.

Polar cod not only show signs of acclimation, though: in the cold, we observed SMR to be actively up-regulated between 55 and 40% air saturation whilst MMR declined steadily, which progressively narrowed AS. Only below 40% air saturation, SMR and MMR followed an oxyconforming pattern with decreasing *P*O_2_ before merging at *P*_crit_. A key question arises from the observation that the calculated maximum oxygen supply capacity (α) at 10 °C was located in the already decreasing part of the MMR curve. This is likely a consequence of an increased oxygen extraction efficiency during decreasing oxygen availability, which could potentially provide a strategy to counteract the onset of anaerobic metabolism. An enhanced oxygen uptake was also observable in SMR. In the absence of additional oxygen demand caused by activity, it is apparent that an elevated metabolic rate was documented within the range of 55% to 40% air saturation, the range of *P*_cmax_ (37.22 ± 12.92% air saturation at 10 °C, see also Fig. 2). This elevated oxygen supply enables the support of MMR at decreasing oxygen concentrations, thereby counteracting the decline in O_2_ availability, resulting in a less pronounced decrease in MMR relative to the decline in *P*O_2_. Consequently, α_0_ = MMR/*P*O_2_ reaches its maximum at lower *P*O_2_ than expected from MMR alone, resulting in a shift in the *P*_cmax_ to the left.

Typically, $\dot M$O_2_ would decline below SMR at *P*_crit_ when fish enter their anaerobic “scope for survival” [70]. However, $\dot M$O_2_ never went below SMR in Polar cod before they lost equilibrium at 9.20 ± 1.80% air saturation in the cold and 27.40 ± 4.20% air saturation in the warmth respectively. We therefore conclude that Polar cod have a very limited anaerobic capacity. In line with this assumption is the decrease of anaerobically fuelled burst swim behaviour with decreasing oxygenation (c.f. burst counts per minute, Table S1), which appears counterintuitive at first sight. This goes hand in hand with a decrease in active swimming duration. Kunz et al. [29] already noted that Polar cod showed only rare and very brief periods of burst swim activity under different temperature/hypercapnia treatments and determined an anaerobically fuelled swimming capacity of only 0–5.7%. Earlier studies have reported a generally low anaerobic capacity for polar fish [94, 95]. Our present data point in the same direction and corroborate the notion that, in Polar cod, these short bouts of anaerobic burst swimming are only possible under conditions where almost instantaneous aerobic recovery of white swimming musculature is possible [cf. 96]. Thus, there are clear indications for a metabolic strategy in Polar cod to maintain aerobic capacity until very low *P*O_2_, instead of allowing for anaerobic metabolism under hypoxic conditions. In comparison to Antarctic stenothermic fish, this finding does not appear to be particularly surprising, and may be attributed to a consequence of cellular metabolic trade-offs, as previously discussed by Pörtner [97]. Antarctic stenothermic fish, particularly those belonging to the notothenioids, have evolved highly efficient aerobic metabolic systems supported by a low-energy-turnover/low-activity lifestyles in cold environments. This adaptation involves an increase in mitochondrial densities and capacities in order to support oxidative metabolism, but this in turn reduces the space available for myofilaments, resulting in lower muscular force production [97]. Consequently, these fish exhibit optimal performance during prolonged, low-intensity activities, yet their capacity to generate high force or engage in intense activities is constrained, as is reflected in the relatively low U_crit_ for Polar cod in the present study. Their reliance on aerobic metabolism diminished their anaerobic capacity and further underscores their adaptation to a low-activity lifestyle in a cold environment, where the demand for energy efficiency prevails over the ability to sustain high-intensity bursts [cf. 97]. However, studies have demonstrated that Polar cod exhibits a lower degree of stenothermy in comparison to Antarctic species. In a transcriptomic study, Kempf et al. [89] demonstrated the absence of a pronounced heat stress response at elevated temperatures of 8 °C and observed a relatively flat and stagnant growth curve in the temperature range between 6 and 8 °C [89]. This is not consistent with the growth curves of other stenothermic species, which typically follow a normal distribution and are characterized by a narrow optimum [cf. 98]. When compared to other fish, such as European seabass (*Dicentrarchus labrax*), other gadoids (e.g. *Gadus morhua, Gadus ogac*), common sole (*Solea solea)* and turbot (*Psetta maxima*) [reviewed by 82], which all display declining SMR and AS with decreasing oxygen content, the regulatory capacity to maintain AS to such low *P*O_2_ levels therefore appears extraordinary in Polar cod. For example, SMR of arctic Greenland cod starts to oxyconform around 45% air saturation [70 mmHg 53], falling below its temperature specific SMR, while Polar cod can increase oxygen extraction and thus $\dot M$O_2_ in this range and never fall below SMR. A precondition for this increase presumably is the exceptionally low SMR, which is among the lowest $\dot M$O_2_ that have been measured in fish. This suggests that relatively little energy is required for oxygen extraction and perfusion in oxygen-rich polar waters, which may be supported by a certain degree of cutaneous respiration that is frequently found in polar fish [e.g. 99–101]. Increased O_2_ extraction costs under progressive hypoxia are evidenced by rising metabolic rates below 50% air saturation. This intermediate increase in routine $\dot M$O_2_ appears as a species-specific strategy to deal with decreasing oxygen levels, and results in one of the lowest *P*_crit_ we are aware of for fish at their respective habitat temperature [Pcrit 2.12 kPa, cf. 74]. Consequently, MMR (and thus aerobic scope) is supported down to relatively low *P*O_2_, which may be of even greater importance for Polar cod in everyday life.

After warm acclimation, we observed similar patterns in SMR and MMR compared to 2 °C, albeit more compressed and with clear signs of increased metabolic costs. Below T_crit_, Q10 would predict a doubling of baseline metabolic rate between 2 and 10 °C, if metabolic compensation during acclimation is disregarded. In the present experiment, the 10 °C acclimated fish displayed a more than 5-fold higher SMR compared to 2 °C, indicating that there was no metabolic compensation during acclimation and that metabolic costs had risen exponentially instead. These high costs can be due to metabolic/mitochondrial inefficiency above Polar cod’s metabolic and growth optima on the one hand [28, 31], and rising costs of the circulatory system on the other [cf. 102]. We found MMR to be increased two-fold in the warmth compared to 2 °C, which can be an acute effect of temperature or a result of acclimation to 10 °C (Fig. 2C and D, Table S1). In comparison to the observed 5-fold rise in SMR, this indicates a physical limitation of either oxygen uptake and cardio-vascular distribution, or ATP turnover and demand. As active swimming time and U_crit_ were not reduced at elevated temperatures compared to 2 °C, and AS was 20% higher in the warmth (between 100 and 50% air saturation), limitations in oxygen distribution and ATP supply are unlikely. We thus conclude that the swimming ability is identical at both temperatures at a 20% increased cost in the warmth.

The higher initial MMR in the warm acclimated fish also led to an increase of absolute AS in the range between 100 and 50% air saturation (Fig. 2F, Table S1). However, comparing absolute AS at different temperatures can be unrealistic, as it ignores rising baseline metabolic costs that claim a higher percentage of metabolic scope [103]. These reflect generally higher maintenance costs in the warmth. Therefore, factorial aerobic scope (MMR/SMR; FAS, Table S2) sinks to < 50% in the warm acclimated fish compared to that at 2 °C, despite the increased absolute AS (Fig. 2E and F). This follows that warm AS is initially larger at 100% air saturation but also starts to decrease earlier (50% vs. 30% air saturation), meeting with cold AS around 65% air saturation (Fig. 2E and F) and running in parallel along an oxyconforming line until finally reaching *P*_crit_ at 22% air saturation. Despite lower FAS, active swimming time was constantly high throughout the oxygen range as opposed to a reduction in active swimming time with decreasing *P*O_2_ levels at 2 °C.

As a proxy for *B. saida*’s anaerobic capacity, we analysed behavioural changes under swimming exercise paired with progressive hypoxia. Its burst swimming behaviour suggests that *B. saida* has a generally low capacity for anaerobic swimming, whereas the overall swimming capacity is comparable to its larger relative and invading predator, *Gadus morhua*, which reaches similar maximum swimming speeds at 5 °C [cf. 104–106]. Interestingly, U_crit_ remained constant over a far longer *P*O_2_ range than AS could be maintained, visibly decreasing only at 10% and 30% air saturation in the cold and warm treatments, respectively. This corresponds to about 20% of total AS in the cold and 35% in the warmth. As such, swimming is compromised only very late when aerobic scope is reduced during progressive hypoxia, indicating that it has a high priority in energy allocation in Polar cod. This indirectly also indicates that not more than 20–35% of aerobic scope is fuelled into swimming, which translates into a relatively modest cost of transport at these temperatures [107]. These data also indicate that cost of transport is lower in the cold for Polar cod, as higher swimming speeds can be realized at lower metabolic rate (cf. Table S1). This is consistent with additional observations in our laboratory, which report an almost linear increase in MR/swimming speed with temperature in the range of 2 to 8 °C (Mark, Kuchenmueller, et al., in prep).

### Ecological & environmental context

At first sight, the presence of a strongly developed hypoxia tolerance in a polar fish may appear counterintuitive, in light of the general paradigm that hypoxic water layers are mostly associated with temperate and tropical waters. Nonetheless, Polar cod can experience hypoxic conditions in the Svalbard fjord systems and may even be evolutionarily adapted to them: as pointed out above, high-silled, semi-closed fjords depend on winter circulation driven by ice formation for bottom water exchange and re-oxygenation, leading to local hypoxia in these deep cold fjord basins where Polar cod spend the summer months [11, 13]. Furthermore, Polar cod is known to occur in very large schools of up to 900 million individuals [26, 108, 109], and generally, schooling leads to progressive hypoxia towards the centre of the schools [110–113]. Polar cod have thus been subjected to an evolutionary need to develop maximised oxygen extraction capacities to reduce ventilatory and general metabolic costs, which is also reflected in a low *P*_crit_.

However, the pertinent question remains whether the observed responses can be summarized under classic hypoxia tolerance, as we a) did not observe any metabolic downregulation and b) no anaerobic component of the hypoxia response in Polar cod, both of which are usually put forward in the definition of hypoxia tolerance [77, 79, 114]. Therefore, we would tend to describe the observed metabolic response to hypoxia rather as metabolic hypoxia compensation than hypoxia tolerance. Both strategies exist to different extents in all species, the balance between tolerance and compensation is what differs across species [115, 116]. While some species may rely more on reducing their oxygen demands (tolerance), others may rely on enhancing their oxygen uptake (compensation) when faced with hypoxia. In the case of the Polar cod, the compensatory response appears to be more pronounced, as the mechanisms involved here actively seek to improve oxygen supply instead of (anaerobically) tolerating hypoxia through metabolic depression. One adaptation found in this study was the upregulated SMR at *P*_cmax_, possibly increasing oxygen extraction efficiency to support metabolism during conditions of low oxygen concentrations, as demonstrated by the observed consistent maximum swimming and gait transition speeds without pronounced burst swimming contribution, even at oxygen levels approaching *P*_crit_ (12.56 ± 1.59% air sat. at 2 °C and 24.37 ± 16.65% air sat. at 10 °C). Polar cod, therefore, may possess unique physiological adaptations that make them more efficient at extracting and utilizing oxygen, even in hypoxic conditions. This could involve a more efficient oxygen-binding capacity of haemoglobin [cf. 117, 118], changes in gill morphology [cf. 119, 120], or a higher density of mitochondria, similar to other cold-acclimated and -adapted fish [121], which would facilitate cellular respiration in low-oxygen environments. Exploring the presence of these mechanisms in Polar cod in future studies could reveal the underlying processes that enable these fish to maintain their SMR under hypoxic conditions. The evolution of this metabolic compensation strategy was possible in the generally cold and oxygen rich Arctic waters, but also necessary, as the Arctic regions around Svalbard underwent several climate warming phases in their past, with winter mean air temperatures being up to 10 K warmer than nowadays [122], with ice free winters and hypoxic fjord bottom waters as a possible consequence. Over the past 100 years, Isfjorden (the connecting fjord between Billefjorden and the ocean) has warmed by 1.9 K [123], Arctic surface air temperatures are projected to rise by 5–10 K [6, 124] and Arctic sea surface temperatures are projected to rise by up to 0.5 K per decade [125].

In light of the ongoing climate change, the question arises whether Polar cod can compensate for warming and hypoxic conditions stronger than the warm phases of the past? While our findings suggest that Polar cod exhibit a considerable tolerance to acute hypoxia, the combined impact of ocean warming and associated ecological shifts may challenge this resilience. Examining the long-term and/or combined effects of hypoxia on the metabolism and other physiological factors, such as growth and reproduction, would help to provide a comprehensive understanding of the long-term resilience of this important species. For a polar fish species, Polar cod displays an extraordinary capacity to endure prolonged periods of elevated temperatures, but it is doubtful that adaptation is fast enough to hold out against rapid climate change.

## Electronic supplementary material

Below is the link to the electronic supplementary material.

Supplementary Material 1

## Data Availability

Raw data are available in the scientific database PANGEA under 10.1594/PANGAEA.962684 [126].
