# Supplementary material for: The physiological response of the Arctic key species Polar cod, Boreogadus saida, to hypoxia in a warming ocean: critical oxygen levels and swimming performance
Source: BMC Zool. 2025 Oct 11;10:20. doi: 10.1186/s40850-025-00241-3 (PMC12514829; doi:10.1186/s40850-025-00241-3)
Supplement: Supplementary file 1 — Supplementary Material 1 [file 40850_2025_241_MOESM1_ESM.docx]

**Supplementary figures and tables from the main manuscript Kempf et al. (DOI: 10.1186/s40850-025-00241-3)**

**Table S1 Summary of the main results from both temperature treatments**

The first columns show the results of the RMR measurements (respiration chambers) and include the ambient oxygen saturation in % air saturation of each *P*O_2_ level, the temperature specific SMR, mean R*Ṁ*O_2_ and SMR for each *P*O_2_ level (in µmol O_2_/g·h). To summarize the AMR (swim tunnel) experiments, the ambient oxygen saturation in % air saturation of each *P*O_2_ level, the mean A*Ṁ*O_2_ and MMR (in µmol O_2_/g·h), U_gait_, U_crit_ (in BL/sec), net cost of transport at U_crit_ (COT_net_= (MMR-SMR) ·0.47 (J/µmol O_2_)/U_crit_; J/km·g), and active swimming during the trial (in min) are listed in the second half of the table. As link between routine and active metabolism, the FAS and AS (as mean of each *P*O_2_ level, in µmol O_2_/g·h) are summarized in the last columns. Due to the randomization of the use of the animals, the metabolic rates for calculating AS and FAS were pooled, resulting in a mathematical artefact of negative values. These have now been removed as incorrect, this affected treatment 30 - 20 % air sat. at 10 °C

**Table S2 Summary of the thresholds and maxima of both temperature treatments**

*P*_crit_ and *P*_crit-max_ of both temperature treatments are listed and converted from % air saturation to µmol/L, and kPa, as well as the temperature specific SMR, maximum MMR, AS and FAS measured along progressing hypoxia (the corresponding *P*O_2_ level in brackets). The last column rates the temperature effect between 2 and 10 °C for the listed parameters. ↑↑↑: strong increase (>3.5-fold), ↑↑: moderate increase (2 - 3.5-fold)., ↑: small increase (<1.5-fold), ↔: no effect (< 0.5-fold), ↓↓: moderate decrease (>1
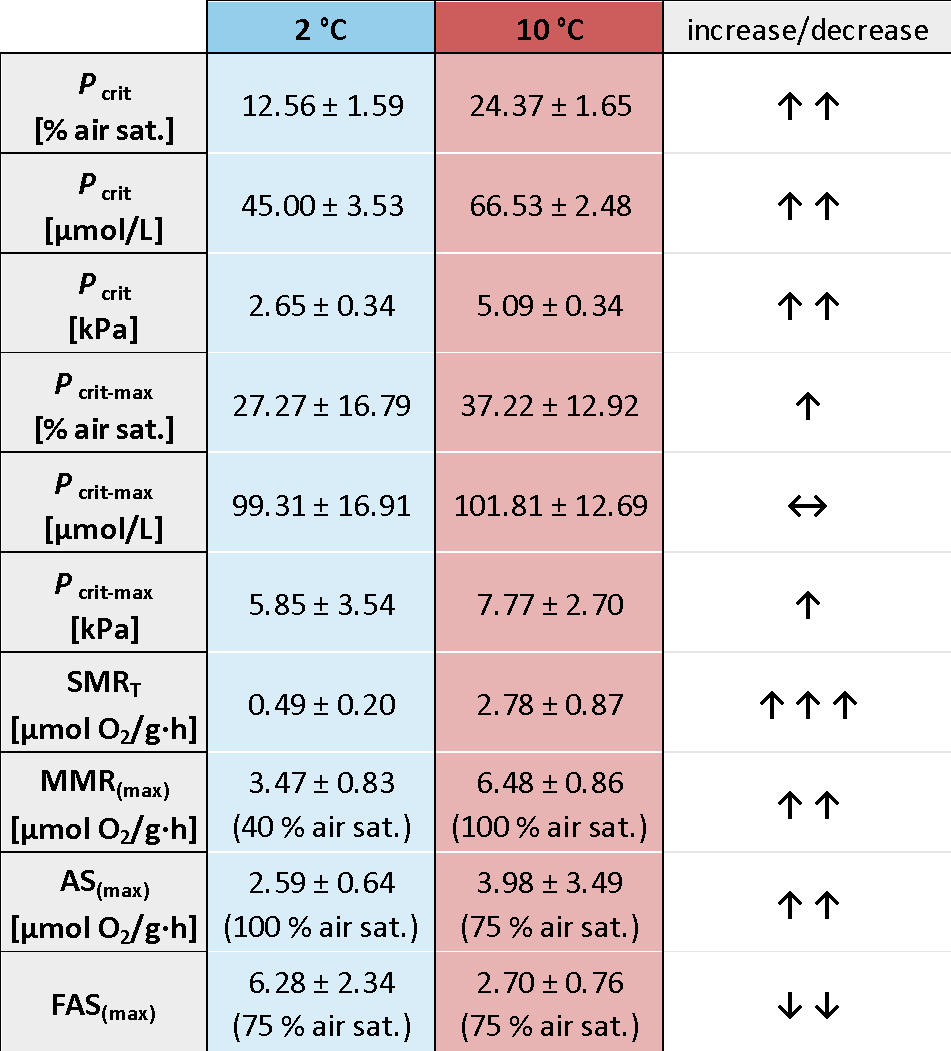
5 - 3.5-fold).

**Table S3 Summary of the mean oxygen concentrations from both temperature treatments**


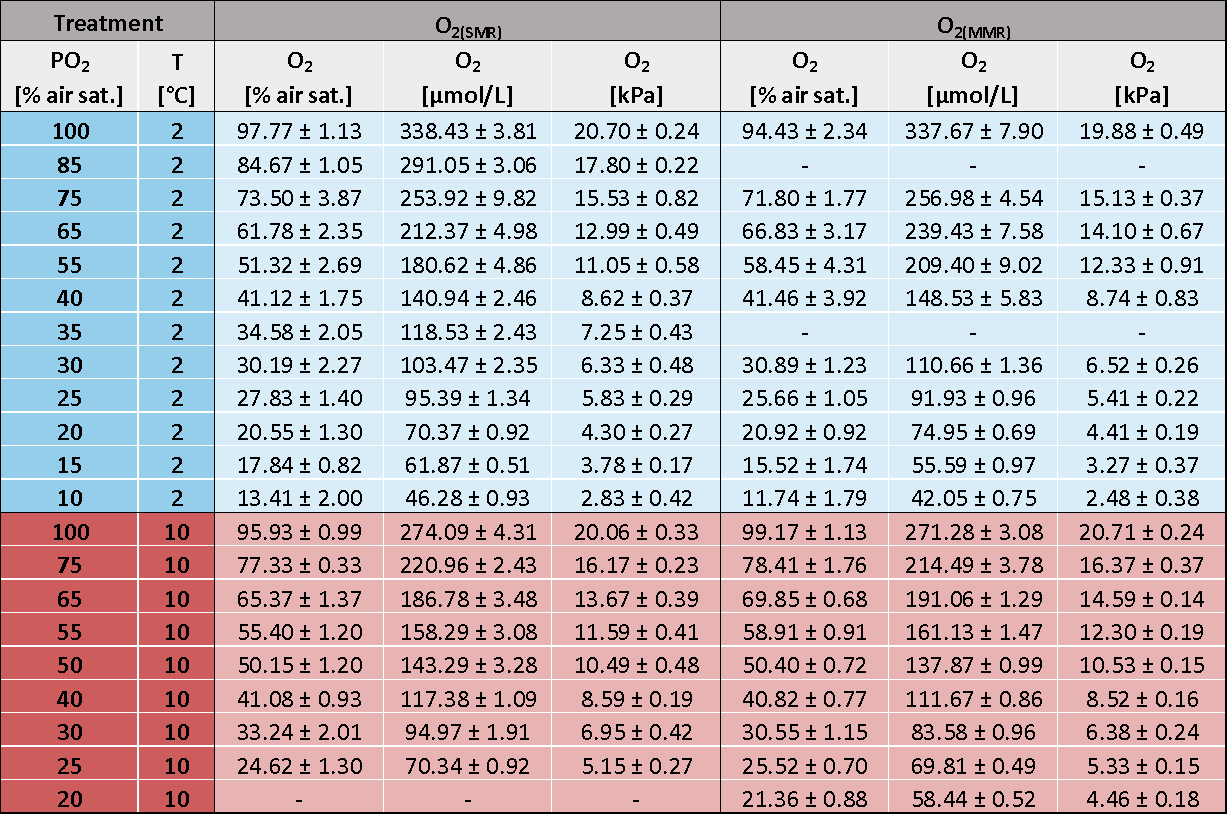
The mean oxygen saturation of each *P*O_2_ level for SMR and MMR are listed and converted from % air saturation to µmol/L, and kPa.

**Table S4 Fish tested at 2 °C (group C)**


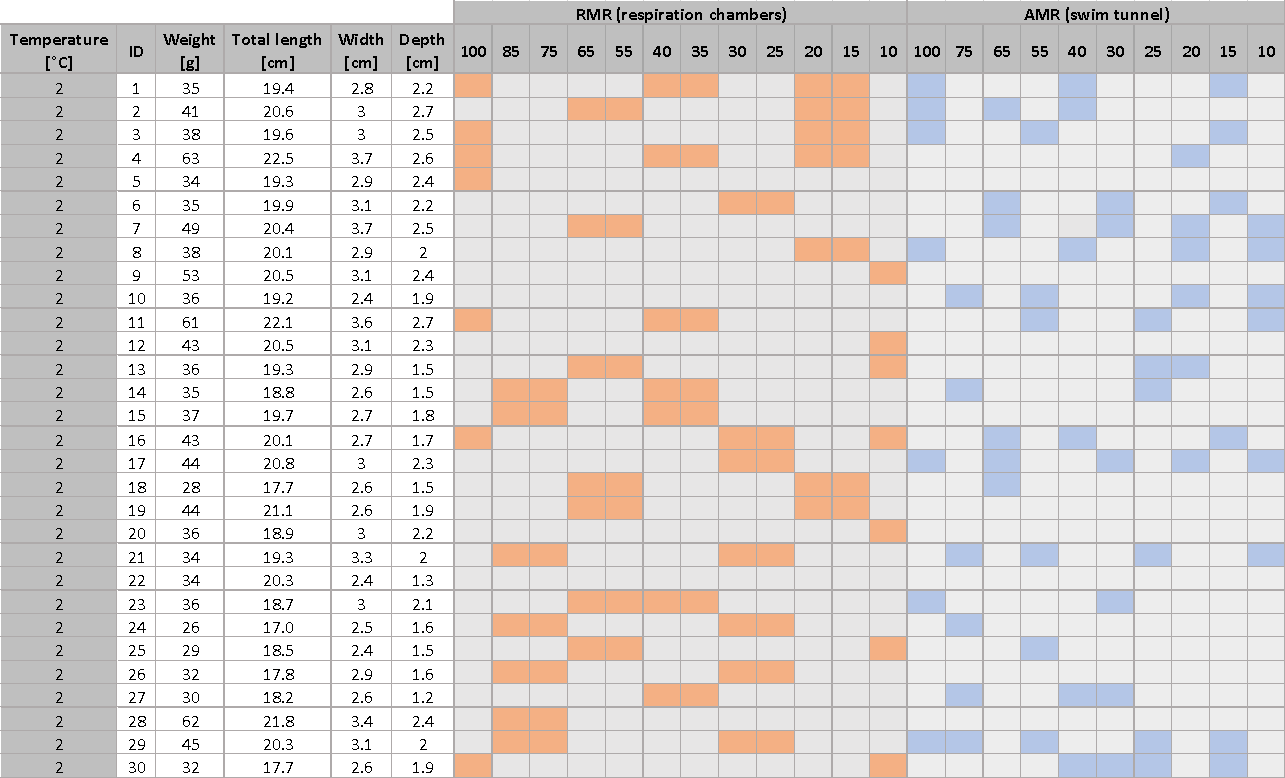
The first columns contain the individual ID-number of each fish and their body parameters: weight (in gram wet weight), total length (cm), width (cm, measured at the thickest part of the body), and depth (cm, measured between dorsal and pelvic fin, highest part of the fish). The following colour code displays the use of each fish during either SMR trials (measurements in respiration chambers, colour code orange if applicable) and/or MMR trials (swim tunnel measurements, colour code blue if applicable). The experiments were conducted in the sequence outlined in the table below: First RMR measurements were taken from the highest to the lowest air saturation, followed by the tests in the swimming tunnel, also from the highest to the lowest oxygen saturation.

**Table S5 Fish tested at 10 °C (group WA)**


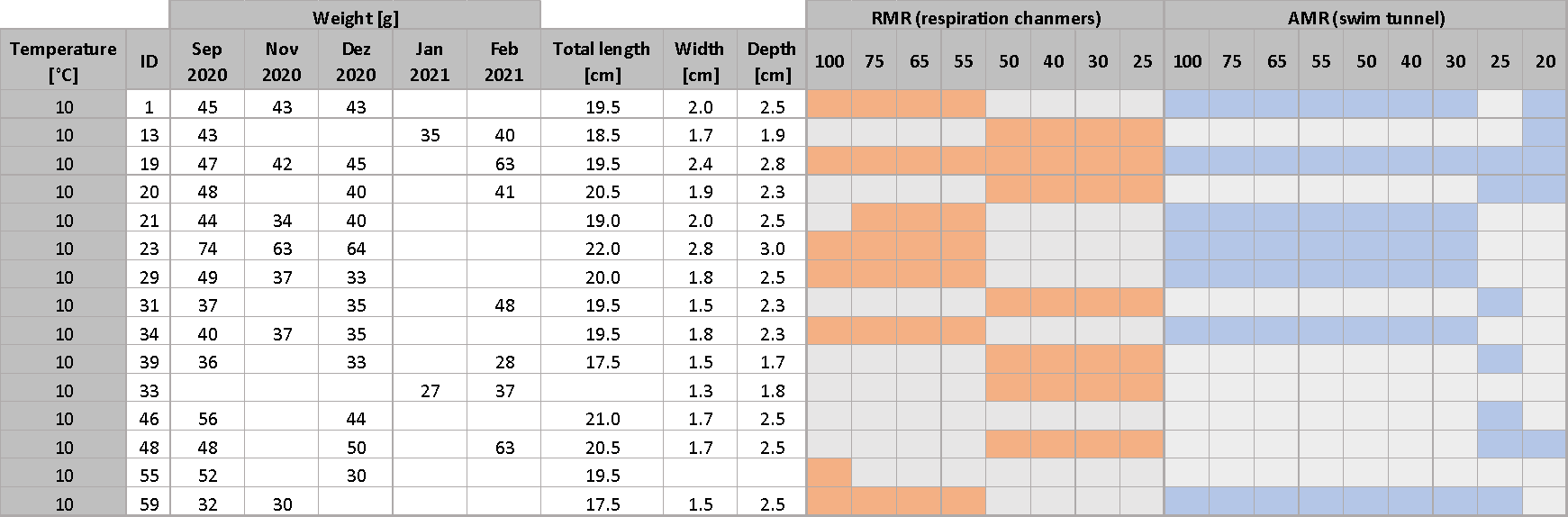
The first columns contain the individual ID-number of each fish and their body parameters: weight (in gram wet weight, measured frequently since the extreme temperature treatment caused weight-loss), total length (cm), width (cm, measured at the thickest part of the body), and depth (cm, measured between dorsal and pelvic fin, highest part of the fish). The following colour code displays the use of each fish during either SMR trials (measurements in respiration chambers, colour code orange if applicable) and/or MMR trials (swim tunnel measurements, colour code blue if applicable). The experiments were conducted in the sequence outlined in the table below: First RMR measurements were taken from the highest to the lowest air saturation, followed by the tests in the swimming tunnel, also from the highest to the lowest oxygen saturation.


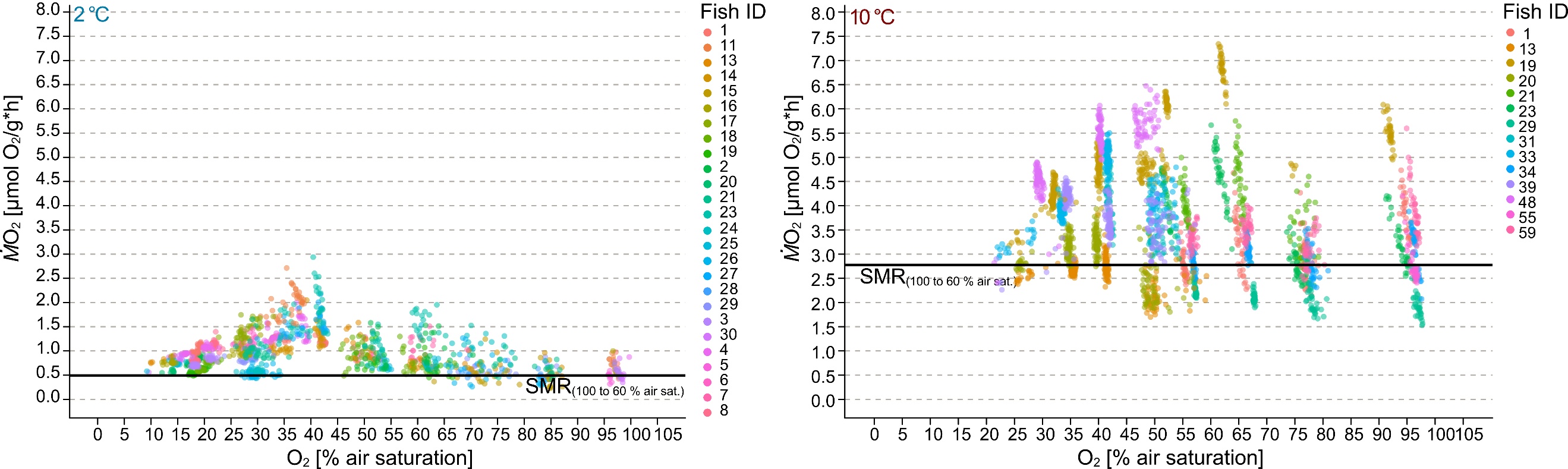
**Figure S1 Additional information to figure 2A and B: Routine metabolism and standard metabolic rate with progressing hypoxia at 2 and 10 °C**

In addition to figure 2, the oxygen consumption (*Ṁ*O_2_ in µmol O_2_/g∙h) over oxygen saturation (in % air saturation) is displayed and each fish is indicated by an individual colour. For both temperature treatments, the temperature specific SMR has been calculated from RMRs measured between 60 and 100 % air saturation (black line). **Left:** *Ṁ*O_2_ at 2 °C. **Right:** *Ṁ*O_2_ at 10 °C.

**
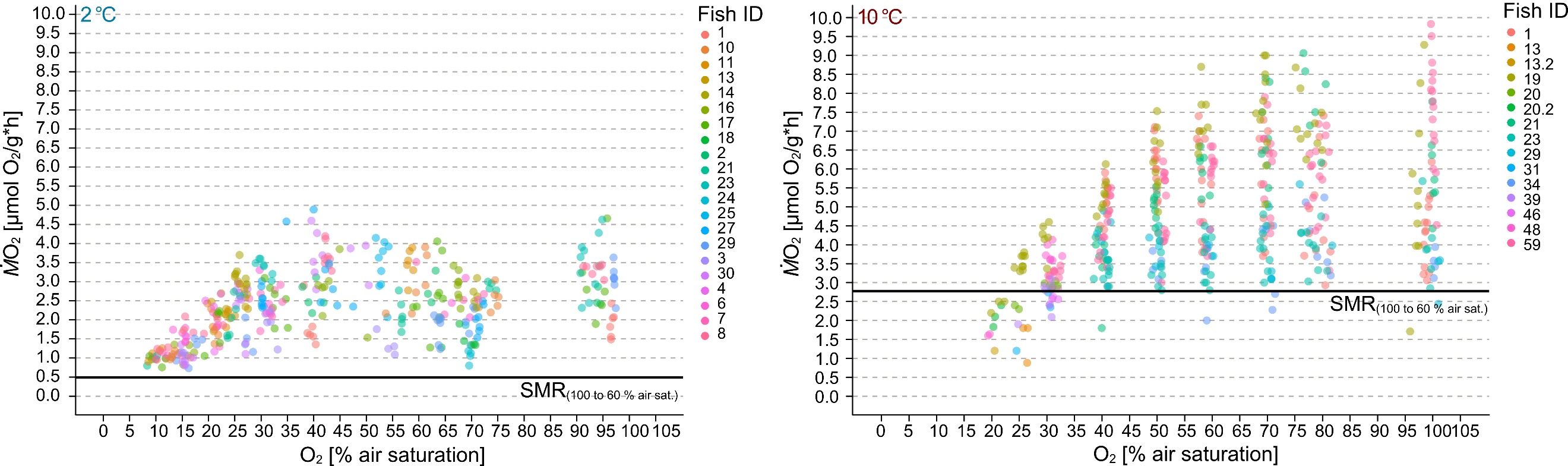
**

**Figure S2 Additional information to figure 2C and D: Active metabolism and maximum metabolic rate with progressing hypoxia at 2 and 10 °C**

In addition to figure 2, the oxygen consumption (*Ṁ*O_2_ in µmol O_2_/g∙h) over oxygen saturation (in % air saturation) is displayed and each fish is indicated by an individual colour. For both temperature treatments, the temperature specific SMR has been calculated from RMRs measured between 60 and 100 % air saturation (black line). **Left:** *Ṁ*O_2_ at 2 °C. **Right:** *Ṁ*O_2_ at 10 °C.


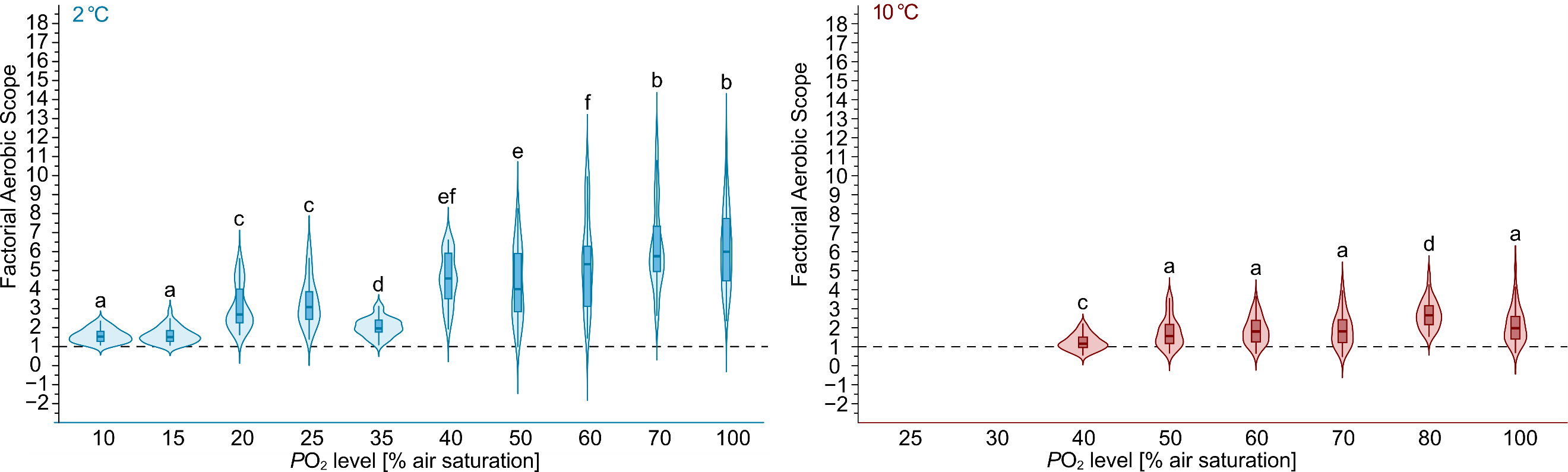


**Figure S3 Factorial aerobic scope (FAS)**

The FAS (MMR/SMR) at 2 °C (left, blue) and 10 °C (right, red) over the *P*O_2_ levels (in % air saturation) are shown as violin plots. Similar letters indicate no significant difference (*p* > 0.05) among *P*O_2_ level based on results of pairwise t-test. Due to the randomization of the use of the animals, the metabolic rates for calculating FAS were pooled, resulting in a mathematical artefact of negative values. These have now been removed as incorrect, this affected treatment 30 and 25 % air sat. at 10 °C


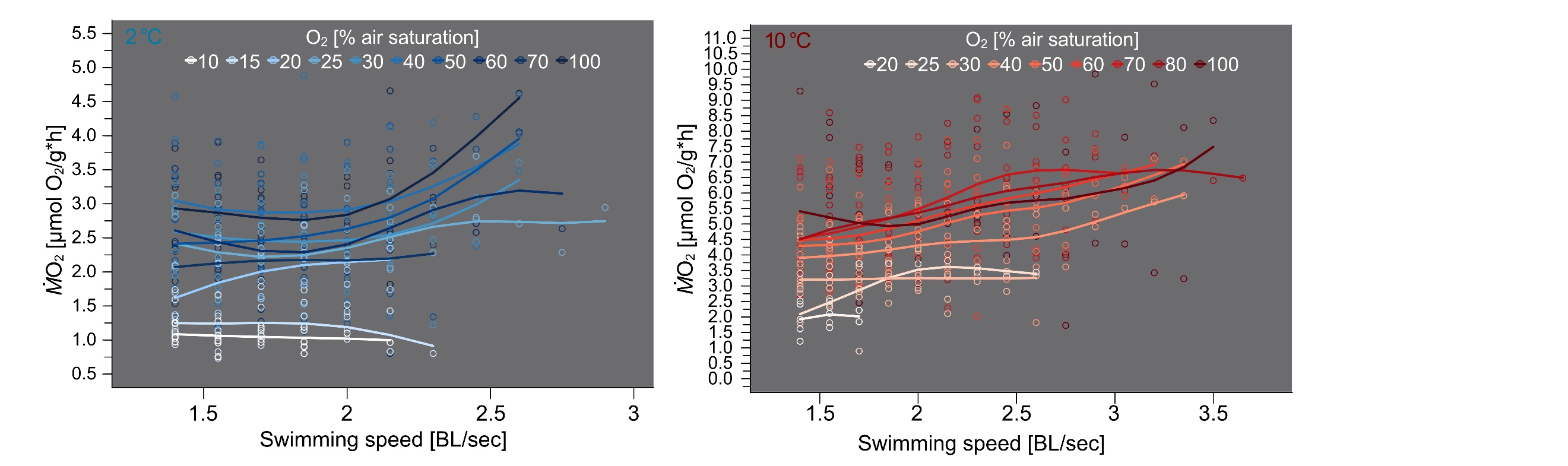


**Figure S4 *Ṁ*O_2_ and water velocity**

*Ṁ*O_2_ (in µmol O_2_/g∙h) is displayed over swimming speed (in BL/sec) for both temperatures (left, blue: 2 °C, right, red: 10 °C). Each line corresponds to the mean *Ṁ*O_2_ for each *P*O_2_ level, circles in the same colour depict individual *Ṁ*O_2_.
